# Predictive and prognostic factors of efficacy of third-line chemotherapy in patients with unresectable pancreatic cancer: a cohort-based study

**DOI:** 10.1093/oncolo/oyaf125

**Published:** 2025-06-14

**Authors:** Camille Evrard, Antoine Pelras, Simon Rivet, Jean-Baptiste Bachet, Olivier Dubreuil, Anne-Laure Pointet, Julien Taieb, Widad Lahlou, Alix Portal, Céline Lepère, Thierry Lecomte, Romain Chautard, Nicolas Williet, Jean-Marc Phelip, Clélia Coutzac, Emilie Soularue, Lysiane Marthey, Raëf Abdallah, Anne Thirot Bidault, Pascal Artru, Jérome Desrame, Bertrand Le Roy, Marine Jary, Pascal Hammel, Isabelle Trouilloud, Nelson Lourenco, Vincent Hautefeuille, Laëtitia Dahan, Simon Pernot, Dominique Béchade, Astrid Pozet, Franck Bonnetain, Christophe Locher, Johann Dréanic, Romain Coriat, Bélinda Tchoundjeu, Yohann Foucher, David Tougeron

**Affiliations:** Medical Oncology Department, Poitiers University Hospital, Poitiers 86000, France; ProDicET, UR 24144, University of Poitiers, 86000 Poitiers, France; Direction of research and Innovation, Platform for Methodology and Biostatistics, Poitiers University Hospital, Poitiers 86000, France; Hepato-Gastroenterology Department, Poitiers University Hospital, Poitiers, France; Sorbonne University, Department of Hepato-Gastroenterology, Pitié-Salpêtrière Hospital, Paris, France; Sorbonne University, Department of Hepato-Gastroenterology, Pitié-Salpêtrière Hospital, Paris, France; Department of Gastroenterology and Gastrointestinal Oncology, Hôpital Européen Georges-Pompidou, AP-HP, Université de Paris, Paris, France; Department of Gastroenterology and Gastrointestinal Oncology, Hôpital Européen Georges-Pompidou, AP-HP, Université de Paris, Paris, France; Department of Gastroenterology and Gastrointestinal Oncology, Hôpital Européen Georges-Pompidou, AP-HP, Université de Paris, Paris, France; Department of Gastroenterology and Gastrointestinal Oncology, Hôpital Européen Georges-Pompidou, AP-HP, Université de Paris, Paris, France; Department of Gastroenterology and Gastrointestinal Oncology, Hôpital Européen Georges-Pompidou, AP-HP, Université de Paris, Paris, France; Department of Gastroenterology and Gastrointestinal Oncology, Tours University Hospital, Tours, France; Department of Gastroenterology and Gastrointestinal Oncology, Tours University Hospital, Tours, France; Department of Gastroenterology, CHU Saint-Étienne, Saint-Etienne, Rhône-Alpes, France; Department of Gastroenterology, CHU Saint-Étienne, Saint-Etienne, Rhône-Alpes, France; Medical Oncology Department, Leon Berard Center, Lyon, France; Department of Gastroenterology, Kremlin Bicêtre Hospital, Assistance publique-Hôpitaux de Paris (AP-HP), Paris Sud University, Le Kremlin Bicêtre, France; Department of Gastroenterology, Kremlin Bicêtre Hospital, Assistance publique-Hôpitaux de Paris (AP-HP), Paris Sud University, Le Kremlin Bicêtre, France; Department of Gastroenterology, Kremlin Bicêtre Hospital, Assistance publique-Hôpitaux de Paris (AP-HP), Paris Sud University, Le Kremlin Bicêtre, France; Oncology Institute Paris Sud, Hopital Privé d’Antony, France; Department of Gastroenterology and Digestive Oncology Jean Mermoz Hospital, Lyon, France; Department of Gastroenterology and Digestive Oncology Jean Mermoz Hospital, Lyon, France; Department of Digestive Surgery and Oncology, Clermont-Ferrand University Hospital, France; Department of Digestive Surgery and Oncology, Clermont-Ferrand University Hospital, France; Digestive and Medical Oncology Department, Hôpital Paul Brousse and University Paris-Saclay, Villejuif, France; Department of Medical Oncology, Saint-Antoine Hospital, Paris, France; Gastroenterology Unit, Saint-Louis Teaching Hospital, Paris, France; Department of Gastroenterology, Amiens-Picardie University Hospital, Amiens, France; Department of Gastroenterology, University Hospital La Timone, Aix-Marseille University, Marseille, France; Department of Oncology, Institut Bergonié, Bordeaux, France; Department of Oncology, Institut Bergonié, Bordeaux, France; Methodology and Quality of Life in Oncology Unit, (Inserm UMR 1098), Besançon University Hospital, Besançon, France; Methodology and Quality of Life in Oncology Unit, (Inserm UMR 1098), Besançon University Hospital, Besançon, France; Department of Gastroenterology, Meaux Hospital, Meaux, France; Gastroenterology and Endoscopy Unit, Cochin Hospital, Assistance Publique-Hôpitaux de Paris (AP-HP), Paris Descartes University, Sorbonne Paris Cité, Paris, France; Gastroenterology Department, Hôpital Cochin, Paris, France; Department of Gastroenterology and Digestive Oncology, Orleans Regional Hospital (CHRO), Orleans, France CI; Direction of research and Innovation, Platform for Methodology and Biostatistics, Poitiers University Hospital, Poitiers 86000, France; CI,C 1402, Inserm, Poitiers University, Poitiers University Hospital, Poitiers 86000, France; ProDicET, UR 24144, University of Poitiers, 86000 Poitiers, France; Hepato-Gastroenterology Department, Poitiers University Hospital, Poitiers, France

**Keywords:** pancreatic cancer, third-line chemotherapy, predictive factors, cohort

## Abstract

**Background:**

Advanced pancreatic ductal adenocarcinoma (aPDAC) has a poor prognosis with median overall survival (OS) of about 12 months. It is therefore important to explore factors that predict the efficacy of third-line chemotherapy (L3) to identify patients who may benefit from this controversial treatment.

**Methods:**

We conducted a multicenter retrospective cohort-based study of 202 French patients treated for aPDAC who received at least three treatment lines from January 2011 to March 2022. We used penalized Cox regressions to predict progression-free survival (PFS) and OS in patients on L3.

**Results:**

Median age at the start of L3 was 64.3 years old and 63.5% had an Eastern Cooperative Oncology Group Performance Status (ECOG-PS) of 0 or 1. The most frequent regimens for L3 were FOLFIRI (25.2% of patients). Median PFS was 2.2 months, while median OS was 4.2 months. In multivariate models, we identified the following predictors of both PFS and OS: age, sex, surgery for the primary tumor, FOLFIRINOX as the first-line therapy, duration of first and second-line treatments, and for L3: ECOG-PS level, peritoneum, liver and/or lung metastasis and depletion of therapeutic resources. The model incorporating these factors provided acceptable discrimination between event and event-free patients at 6 months post-L3 (area under the ROC curve of 0.83 for PFS and 0.73 for OS).

**Conclusion:**

The characteristics of patients and their aPDAC are readily available in clinical practice and were able to predict survival with L3. The online calculator we propose here could help physicians determine whether L3 chemotherapy would be beneficial.

**Implications for practice**

The efficacy and relevance of third-line chemotherapy in unresectable pancreatic adenocarcinoma remains debated. In this study, median progression-free survival with L3 for unresectable pancreatic cancer was 2.2 months and median overall survival was 4.2 months. In multivariate analysis, long L2 duration and resection of the primary tumor were associated with longer PFS with L3. Predictive models for PFS and OS with L3 allow us to better inform patients and improve shared decision-making.

## Introduction

Pancreatic adenocarcinoma (PA) is the fourth leading cause of cancer-related deaths in the world and is destined to become the second cause in Europe in 2030.^1-3^ PA is mostly diagnosed at an unresectable stage, that is to say locally advanced (30%) or metastatic (50%).^2,4^ Advanced pancreatic ductal adenocarcinoma (aPDAC) has a poor prognosis, with median overall survival (OS) of less than 12 months for metastatic stages (11.1, 11.1, and 8.5 months with standard first-line treatment FOLFIRINOX (Ffx: leucovorin, 5-fluorouracil (5-FU), oxaliplatin and irinotecan), NALIRIFOX and gemcitabine-nab paclitaxel (GNp), respectively).^5-7^ The main prognostic factors for OS in patients treated in a first-line (L1) setting are age, performance status (PS), albumin level, presence of liver metastases, and number of metastatic sites,^5,6^ while other studies have also identified lymph node metastases, lung metastases, carbohydrate antigen 19-9 (CA19-9) level, carcino-embryonic antigen (CEA) level, and/or surgery for the primary tumor.^3,8-10^

About 50% of patients who had L1 chemotherapy are eligible for a second-line treatment (L2) due to the aggressiveness of the disease.^5,6,11,12^ In the retrospective cohort-based study conducted by Taieb et al (*n* = 2565), L2 was given to 64.9% of patients. Patients treated with gemcitabine-based chemotherapy in L1 less frequently had L2 than did patients treated with Ffx (40.5% vs 78.1%).^13^ Until recently, the standard of care in L2 was oxaliplatin-based or irinotecan-based regimens after gemcitabine-based therapy in L1, and gemcitabine-based chemotherapy after Ffx in L1.^14-16^ Following the NAPOLI-1 study, nanoliposomal irinotecan (nal-IRI) in combination with 5-FU and leucovorin (nal-IRI + 5-FU/LV) was approved in several countries, in preference to 5-FU/LV alone, as it increased OS.^14^ Patients who survived more than 1 year were more likely to be aged≤65 years, had a Karnofsky performance status≥90, a neutrophil-to-lymphocyte ratio (NLR) ≤ 5, a CA19-9 level < 59 × the upper limit of normal and were less likely to have liver metastases.^14,17^ Two studies explored predictors of OS in patients with aPDAC on L2, both after L1 with GNp, and showed that a high NLR, a short time-to-progression (TTP) with L1, a high CEA level and a high Glasgow prognostic score (based on serum C-reactive protein and albumin) were independent predictors of poor OS in an L2 setting.^18,19^

In contrast to L1 and L2, third-line (L3) therapy in aPDAC has rarely been studied and there are few data concerning prognostic factors in patients treated with L3. In recent studies, only 5%-30% of patients with aPDAC who had received L1 were able to have L3. In the phase-III GEMPAX L2 study, 47% of patients in the gemcitabine arm and 32% in the gemcitabine plus paclitaxel arm were able to receive L3,^20^ which mainly consisted of taxane for patients in the gemcitabine arm and platinum salt or an irinotecan-based regimen for patients in the gemcitabine plus paclitaxel arm. Few data concerning the efficacy and safety L3 for aPDAC are available.^13,19,21^ In the study by Bachet et al, in which only 24 patients received L3, 16% experienced a partial response (PR), 25% stable disease, and 33% progressive disease (PD) according to Response Evaluation Criteria In Solid Tumors (RECIST) 1.1.^21^ These poor results call into question the relevance of L3. Nevertheless, the prognostic factors of L3 efficacy were not analyzed, even though they could be useful for treatment decision-making.

In this context, the objective of this multicenter retrospective cohort-based study was to describe L3 outcomes in patients with aPDAC to determine related prognostic factors and to propose multivariate predictive tools for L3, the aim being to identify patients with a more favorable prognosis.

## Patients and methods

### Study population

We retrospectively collected data from January 2011 to March 2022 in 15 French centers. Inclusion criteria were all consecutive patients with histologically proven aPDAC (locally advanced unresectable and metastatic), treated with at least three lines of chemotherapy (at least one cycle of L3 treatment). Non-inclusion criteria were patients with tumors other than adenocarcinoma and a tumor other that aPDAC within the 3 years before inclusion.

Our institution’s Ethics Committee waived the need for informed consent because of the retrospective nature of the study and because most patients had died. This study was conducted in accordance with current French law and according to the ethical principles of the Declaration of Helsinki 1975 and its subsequent revisions.

### Patient and tumor characteristics

The following data were collected at diagnosis and at the beginning of L3: patients’ age, sex, weight (kg), height (cm) and body mass index (BMI), Eastern Cooperative Oncology Group Performance Status (ECOG PS), and resection of the primary tumor; blood test results: carbohydrate antigen 19-9 (CA-19-9) and carcinoembryonic antigen (CEA), albumin, bilirubin, alanine aminotransferase (ALT) and aspartate aminotransferase (AST), alkaline phosphatase (ALP), gamma glutamyl transferase (GGT), lactate dehydrogenase (LDH), complete blood count (for NLR) and tumor characteristics (location, node invasion, and metastatic sites).

### Chemotherapy and toxicity

Treatment characteristics were collected for each line of chemotherapy. These included chemotherapy regimens, date of and reasons for ending each line of treatment (progression, toxicity, death, or other reason), number of cycles, treatment efficacy according to RECI,ST 1.1, and the best response obtained for each line. For this study, we defined a new line of treatment as the use of a new drug. Maintenance therapy was not considered a new line of treatment, nor was the re-introduction of the induction regimen (eg, Ffx then 5-FU then Ffx is considered the same line of treatment). The type and grade of toxicities for L3 were collected according to the Common Terminology Criteria for Adverse Events (CTCAE, version 4.0). The depletion of therapeutic resources was defined as patients who had already received 5-fluoruracil, oxaliplatin, irinotean, gemcitabine, and taxane (nab-paclitaxel, paclitaxel, or docetaxel).

### Outcomes

The study baseline was the beginning of L3. Two main outcomes were considered: time to the first event, either death or progression (right censoring at the last follow-up alive without progression to obtain progression-free survival (PFS)), and time to death regardless of the cause and whether there was progression (right censoring at the last follow-up alive to obtain the OS).

### Statistical analyses

Patients’ characteristics at baseline were described using means and standard deviations or medians and interquartile ranges for continuous variables, and numbers and proportions for categorical variables. PFS and OS were described using the Kaplan-Meier estimator^22^ and compared with the Log Rank statistic. We presented the therapeutic sequence using a Sankey diagram done with R Studio freeware (R Foundation for Statistical Computing, Vienna, Austria). We used a proportional hazards model to predict survival,^23^ the baseline hazard function being non-parametrically obtained using the Breslow estimator.^24^ A least absolute shrinkage selection operator (LASSO) was used to select the predictors.^25^ The penalty parameter was estimated by minimizing the cross-validation error at 20 partitions.^26^ The following predictors were considered: age at the start of L3, sex, PFS with L1 and L2, resection of the primary tumor, regimen used in L1, depletion of therapeutic resources, ECOG PS at the start of L3, presence of peritoneal carcinomatosis at the start of L3, liver metastases and lung metastases at the start of L3. We tested the assumptions of log-linearity and proportionality of hazards. To consider missing data on predictors, multivariate chained equation imputation (MICE) was performed to create five complete databases.^27^ MICE assumes missing data are random. This method is used for multivariate data and runs with continuous and qualitative variables. The advantage is that it preserves variability and improves predictive power.

In addition, to avoid over-fitting, bootstrap cross-validation was used with 1000 resamplings.^28^ The contributions of predictors were represented by the percentage of samples in which they were selected among predictors. The following metrics were used to describe predictive capacities: the Brier score at 3 months, the integrated Brier score up to the last observed event, and the area under the ROC (receiver operating characteristic) curve (AUC).^29,30^

## Results

### Patients’ and tumor characteristics at diagnosis

Among the 202 included patients, the median age was 63.1 years, 90.9% were ECOG PS 0-1 and 24.8% had undergone surgery for their primary pancreatic tumor (Supplementary Table S1). The median albumin level was 40.0 [33.0; 43.0] g/L, median bilirubin was 10.0 [5.0; 36.5] Umol/L and median CA 19-9 was 562.0 [75.0; 3273.0] IU/L. The median NLR at diagnosis was 3.3 [2.0; 6.4]. Primary tumors were mainly in the pancreatic head (52.8%), and most patients had synchronous metastasis (69.3%). Most of the patients had metastatic disease at inclusion (69.3%) and most had only one metastatic site at diagnosis (70.0%), namely liver metastasis and peritoneal carcinomatosis in 70.7 and 25.7% of cases, respectively. Ten patients (4.9%) had lung-only metastasis. Median OS since the initial tumor diagnosis was 16.8 months [12.7; 23.7] and 20.4 [16.8; 25.4] and 16.3 [15.3; 17.8] months for patients with and without primary resection of the pancreatic tumor, respectively.

### First and second-line chemotherapy

**Figure 1.**
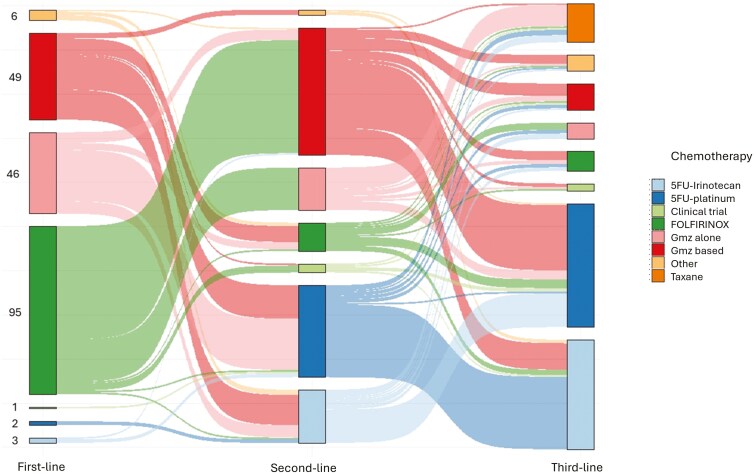
Sankey diagram of treatment sequence, different lines of treatment are shown on the × axis and number of patients on the y axis. Abbreviations: 5 FU:5 fluorouracil; Gmz: gemcitabine

The most frequent first-line chemotherapies were 5FU-based (52.5%), 47.0% of which were the Ffx regimen, followed by gemcitabine-based regimens (47.5%), equally distributed between gemcitabine alone and gemcitabine combined with nab-paclitaxel (Figure 1 and Supplementary Figure S1). The median duration of L1 was 5.1 months. The best response according to RECI,ST 1.1 was stable disease (SD, 38.7%), progressive disease (PD, 35.1%), and complete/partial response (CR/PR, 26.2%). The disease control rate (CR, PR, and SD), all treatment periods considered, was 64.8%. The main reasons for stopping L1 were PD (92.4%) and toxicity (7.1%). Median PFS in L1 was 6.1 months in the overall population and 6.9, 6.5, 3.8, and 2.9 months for patients treated with gemcitabine-based chemotherapy, Ffx, other regimens, and gemcitabine alone, respectively. Median OS with L1 was 16.8 months (Supplementary Figure 2).

The most frequently used L2 regimens were 5FU-based (50.0%), with 23.8% of FOLFOX and 12.4% of FOLFIRI, followed by gemcitabine-based (47.6%) regimens, with 34.7% of gemcitabine plus nab-paclitaxel and 11.9% of gemcitabine alone (Figure 1). The two main therapeutic sequences leading to L3 were: gemcitabine (alone or in combination) followed by 5-FU platinum and then 5-FU irinotecan (*n* = 41, 20.3%) and Ffx followed by gemcitabine (alone or in combination) and then 5-FU platinum (*n* = 39, 19.3%).

The median duration of L2 was 3.1 months. The best response according to RECI,ST 1.1 was PD (53.3%), SD (33.8%), and CR/PR (12.9%). The disease control rate was 46.8%. The main reasons for stopping L2 were tumor progression (95.0%) and toxicity (4.0%). In the overall population, the median PFS with L2 was 3.5 months (Supplementary Figure S3) and median OS with L2 was 9.1 months. There was no correlation between response to L1 and L2 (*P* = .83).

### Patients’ and tumor characteristics at the beginning of third-line treatment

Median age at the start of L3 was 64.3 [57.4; 72.9] years (Table 1) and median BMI was 21.6 [19.2; 25.1]kg/m^2^. ECOG-PS was 0-1 for 63.5% of patients and 2 for 33.7%. Most patients (94.5%) had lost weight with a median loss from L1 of 16.5 [10.0; 25.0]%. Median CA 19-9 was 805.0 [158.0; 6349.0]IU/L, median CEA was 52.0 [12.0; 163.0]IU/L and median LDH was 358.0 [266.0; 434.0]IU/L. Most patients had 1 or 2 metastatic sites: 37.7% and 36.1%, respectively. Liver metastases were the most frequent (78.3%) followed by peritoneal metastases (43.9%). The median time between the start of L1 and that of L3 was 11.4 [8.4; 16.4] months.

**Table 1.** Patients’ and tumor characteristics at the beginning of L3.

Clinical characteristics	*N* = 202
Age (years, median [Q1; Q3]) Men/women (*n*, %)	64.3 [57.4; 72.9] 103/99 (51.0% / 49.0%)
BMI (kg/m^2^, median [Q1; Q3], NA = 28)	21.6 [19.2; 25.1]
Weight loss between diagnosis and L3 (%, median [Q1; Q3], NA = 30)	16.5 [10.0; 25.0]
ECOG PS (*n*, %, NA = 13)	
0-1	120 (63.5%)
≥ 2	69 (36.5%)
Biological characteristics	
NLR (median [Q1; Q3], NA = 141)	2.9 [1.7; 4.9]
AST (IU/L, median [Q1; Q3], NA = 51)	25.0 [21.0; 38.5]
ALT (IU/L, median [Q1; Q3], NA = 51)	27.0 [20.0; 42.5]
ALP (IU/L, median [Q1; Q3], NA = 49)	154.0 [96.0; 296.0]
GGT (IU/L, median [Q1; Q3], NA = 53)	180.0 [66.0; 431.0]
Total bilirubin (umol/L, median [Q1; Q3], NA = 45)	6.5 [4.0; 11.0]
LDH (IU/L, median [Q1; Q3], NA = 113)	358.0 [266.0; 434.0]
CEA (IU/L, median [Q1; Q3], NA = 66)	52.0 [12.0; 163.0]
CA 19-9 (IU/L, median [Q1; Q3], NA = 62)	805.0 [158.0; 6349.0]
Tumor characteristics Metastatic / locally advanced disease	198 / 4 (98.0 / 2.0%)
Number of metastatic sites (NA = 18, *n*, %)	N = 180
1	68 (37.7%)
> 1	112 (62.3%)
Location of metastasis (NA = 62, *n*, %)	N = 180
Peritoneum	79 (43.9%)
Liver	141 (78.3%)
Lung	66 (36.7%)
Patients with liver metastasis	N = 141
With other metastases	95 (67.4%)
Isolated liver metastases	46 (32.6%)
Prior treatments	
FOLFIRINOX for L1	95 (47.0%)
Depletion of therapeutic resources^*^	73 (36.1%)
Median delay between L1 start and L3 start (months) [Q1; Q3]	11.4 [8.4; 16.4]

ALP, alkaline phosphatase; ALT, alanine aminotransferase; AST, aspartate aminotransferase; BMI, body mass index; CA 19-9, carbohydrate antigen 19-9; CEA, carcinoembryonic antigen; ECOG PS, Eastern Cooperative Oncology Group Performance Status; GGT, gamma glutamyl transferase; IU/L, international units per liter; L1, first-line treatment; L3, third-line treatment; LDH, lactate dehydrogenase;NA, not attributed; NLR, neutrophil to lymphocyte ratio; Q1 and Q3, first and third quartiles; umol/L, 10⁻⁶moles per liter

^*^Defined as a patient who had already received 5-fluoruracil, oxaliplatin, irinotecan, gemcitabine, and taxane at the beginning of L3.

The most frequent regimens for L3 were reintroduction of FOLFIRI (25.2%) or FOLFOX (17.8%) and 5FU-cisplatin (16.9%) (Figure 1 and Table 2). The best response for L3 was PR (2.9%), SD (22.1%), and PD (75.0%). The disease control rate was 25.0%. The main reasons for ending treatment were PD (96.4%), toxicity (2.1%) or chemotherapy holidays (1.5%) (Table 2). There was no correlation between response to L1 and response to L3 (*P* = 0.78), whereas there was a strong correlation between response to L2 and response to L3 (*P* < 0.01).

**Table 2.** L3 treatments and related response.

Treatment regimen	*N* = 202
5FU-irinotecan^*^	62 (30.7%)
FOLFOX	36 (17.8%)
5FU-cisplatin	34 (16.9%)
Taxane^**^	22 (10.9%)
Gemcitabine-based^#^	24 (11.9%)
FOLFIRINOX	11 (5.4%)
Others	13 (6.5%)
**Best response according to RECIST 1.1**	*N* = 172
PD	129 (75.0%)
SD	38 (22.1%)
PR	5 (2.9%)
**Reason for treatment stop**	*N* = 195
Progressive disease	188 (96.4%)
Toxicity	4 (2.1%)
Therapeutic break	3 (1.5%)

FOLFOX (LV5FU2 and Oxaliplatin). 5FU-CDDP (LV5FU2 and cisplatin).

FOLFIRI (LV5FU2 and irinotecan). LV5FU2 (leucovorin and 5Fluorouracil).

Others: 5FU or LV5FU2 and mitomycin C. LV5FU2 and nab-paclitaxel. nivolumab. olaparib. clinical trials or chemoradiotherapy with capecitabine (Supplementary Table S1).

^*^FOLFIRI, 5FU and naliri or XELIRI (capecitabine and irinotecan) and FOLFIRI 3 (irinotecan 100 mg/m^2^ at days 1 and 3 combined with leucovorin 400 mg/m^2^ day 1 and 46-h continuous 5-fluorouracil 2000 mg/m^2^).

^**^Taxane: docetaxel or paclitaxel.

^#^Gemcitabine alone or associated with nab-paclitaxel. capecitabin. oxaliplatin or erlotinib.

### Outcomes of L3

**Figure 2.**
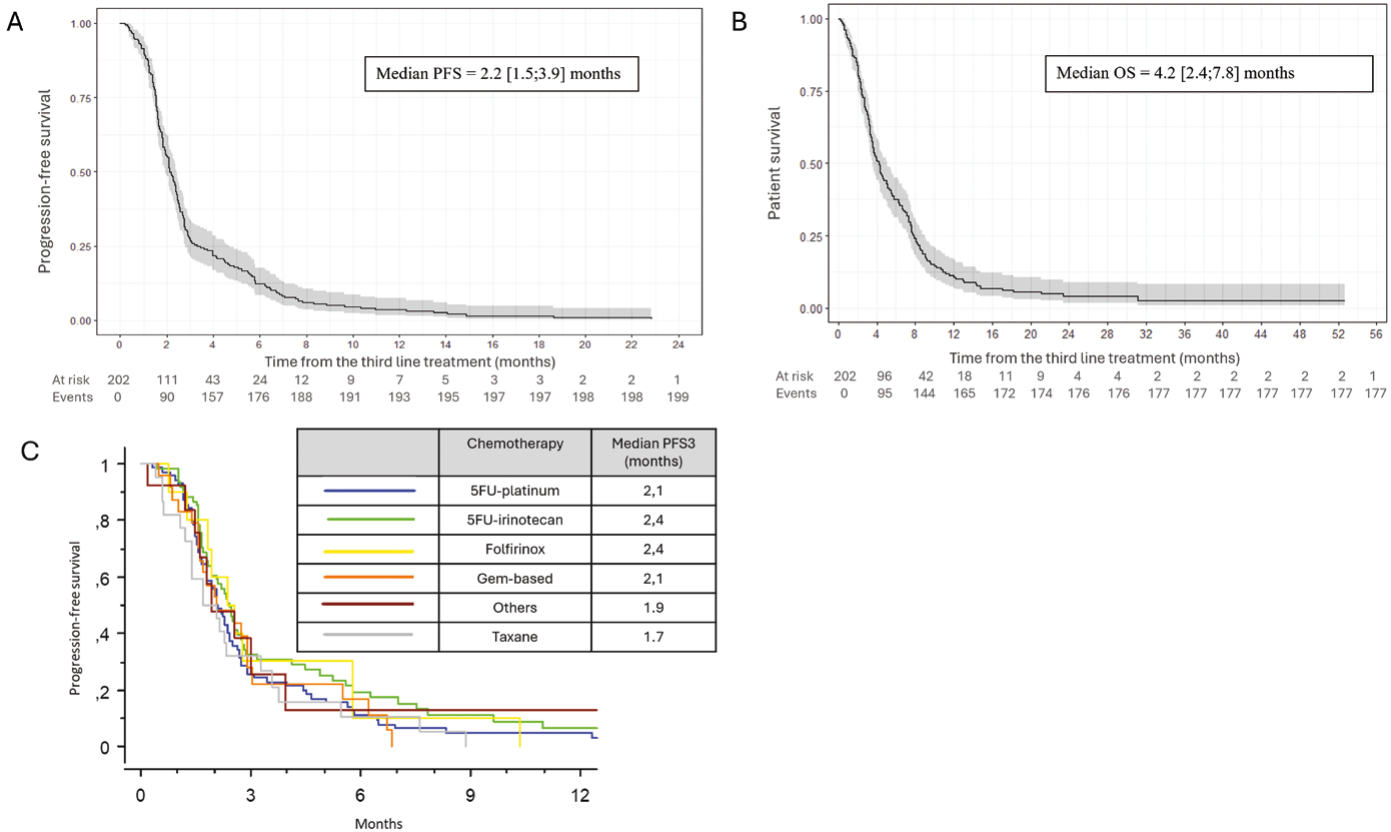
Survival from the start of third-line therapy. (A) Progression-free survival in third-line therapy. (B) Overall survival in third-line therapy. (C) Survival with third-line therapy according chemotherapy regimens. Abbreviations: OS: overall survival; PFS: progression-free survival; PFS3: progression-free survival for third-line treatment

Median PFS in L3 was 2.2 months [1.5; 3.9] (Figure 2A). Median PFS did not significantly differ according to the treatment regimen: 2.4 months for patients treated with FOLFIRI/FOLFIRI3 vs 2.1 months for patients treated with 5FU-cisplatin (Figure 2C). At the end of follow-up, 14 (6.4%) patients were still undergoing L3. Most patients had died (88.1%). Median OS from the beginning of L3 was 4.2 months [2.4; 7.8] (Figure 2B). For patients with primary tumor resection, median OS was 7.6 months [5.1; 8.3] vs 3.7 months [3.3; 4.4] for patients without primary tumor resection.

Regarding safety, 85.6% of patients had at least one adverse event, with 19.9% of grade 3 or 4. The most frequent toxicities were gastrointestinal (28.7%), neuropathy (23.3%), and hematologic (18.6%). No treatment-related deaths occurred.

At progression or at the end of L3, 32.2% of patients received fourth-line chemotherapy (mainly FOLFIRI/FOLFIRI3 for 29.2%), and 67.8% of patients received best supportive care (BSC). The latter died after a median time of 43 days after the end of L3 (range from 0 to 353).

### Predictors of L3 outcomes

In univariate analysis, we identified the following predictors of longer PFS and OS: ECOG PS 0-1 at the start of L3, resection of the primary tumor, no depletion of therapeutic resources at L3, no liver metastasis at L3 and long L2 duration (*P* < 0.05, Supplementary Table S2). Variables with more than 20% missing data were not included in the univariate or multivariate models.

The results of the multivariate analysis are reported in Table 3. Among the main predictors of better PFS, the most significant were long L2 duration (HR = 0.76, 95%CI, 0.60-0.97), resection of the primary tumor (HR = 0.59, 95%CI, 0.39-0.91) and ECOG-PS 0-1 at the start of L3 (HR = 0.53, 95%CI, 0.38--0.75). Those associated with longer OS were L2 duration (HR = 0.62, 95%CI, 0.48--0.81), no depletion of therapeutic resources at the start of L3 (HR = 1.79, 95%CI, 1.10--2.91) and ECOG-PS 0-1 at the start of L3 (HR = 0.56, 95%CI, 0.40--0.79).

**Table 3.** Predictive models for L3 progression-free survival and overall survival

	Progression-free survival HR [IC95%]	Overall survival HR [IC95%]
Age (years)	0.99 [0.98; 1.02]	1.01 [0.99; 1.02]
Sex (male vs female)	1.22 [0.89; 1.67]	1.27 [0.91; 1.76]
Surgery of primary tumor (Yes vs No)	0.59 [0.39; 0.91]	0.68 [0.43; 1.07]
L1 chemotherapy (Folfirinox vs Other)	0.96 [0.63; 1.45]	0.79 [0.50; 1.25]
Depletion of therapeutic resources^*^ (yes vs No)	1.41 [0.91; 2.20]	1.79 [1.10; 2.91]
ECOG PS in L3 (0-1 vs 2-3)	0.53 [0.38; 0.75]	0.56 [0.40; 0.79]
Lung metastasis in L3 (yes vs No)	1.12 [0.76; 1.67]	1.18 [0.79; 1.76]
Peritoneum metastasis in L3 (yes vs No)	1.11 [0.72; 1.70]	1.24 [0.79; 1.93]
Liver metastasis in L3 (reference: with other)	1.05 [0.71; 1.57]	1.40 [0.94; 2.09]
Isolated	1.70 [1.04; 2.78]	0.99 [0.60; 1.65]
No	0.78 [0.51; 1.21]	0.85 [0.54; 1.34]
Logarithm of L1 duration (months)	0.99 [0.82; 1.22]	1.12 [0.90; 1.38]
Logarithm of L2 duration (months)	0.76 [0.60; 0.97]	0.62 [0.48; 0.81]

^*^Defined as a patient who had already received 5-fluoruracil, oxaliplatin, irinotecan, gemcitabine, and taxane at the beginning of L3.

Abbreviations: 95% CI, 95% confidence interval; ECOG PS, Eastern Cooperative Oncology Group Performance Status; HR, hazard ratio; L1, first-line treatment; L3, third-line treatment.

### Prognostic capabilities of the multivariate models

The 2 multivariate models in Table 3 have acceptable discriminative capacities with AUCs for a prognosis up to 6 months from L3 equal to 0.73 (95%CI, 0.65--0.81) and 0.83 (95%CI, 0.75-0.90) for PFS and OS, respectively. The related Brier scores were 0.10 (95%CI, 0.07-0.13) and 0.22 (95%CI, 0.19-0.26), respectively. We found no calibration issues as presented in the calibration plots for PFS and OS at 3 and 6 months (Supplementary Figure S4).

Regarding these results, we propose a freely available online calculator that predicts PFS and OS according to a patient’s characteristics: https://poitiers-health-data.shinyapps.io/KP-L3/. In this calculator, we included the following parameters: sex, age (years), primary resection (yes/no), metastatic site at diagnosis (yes/no), duration of L1 (months), chemotherapy regimen in L1 (Ffx/other), duration of L2 (months), depletion of therapeutic resources at third-line prescription (yes/no), ECOG-PS at L3 (0-1/2-3), lung metastasis at L3 (yes/no), peritoneum carcinosis at L3 (yes/no), liver metastasis at L3 (no/isolated/with other) (Supplementary Figure S5A and B).

## Discussion

In this large retrospective study, we identified the following predictors of L3 efficacy in aPDAC: surgery for the primary tumor, ECOG-PS 0-1, and a long duration of L2. These factors allowed us to build a score that provides a more precise estimation of expected PFS and OS with the third-line treatment. This score could help practitioners, in consultation with their patients, to decide whether or not to administer L3.

To the best of our knowledge, few studies have evaluated factors associated with PFS/OS in L3 settings for aPDAC. Until recently, third-line chemotherapy was seldom administered as only about half of patients were eligible to receive even a second-line treatment because of the deterioration in their general health.^5,6,11,12^ In the NAPOLI-1 study, 31% of patients in the nal-IRI + 5-FU group and 38% in the 5-FU group received L3.^14^ OS was worse in patients treated with L3 or later lines (5.4 and 4.3 months for nal-IRI + 5-FU/LV and 5-FU/LV, respectively) than in patients treated with L1 or L2 (6.2 vs. 4.2 months).^31^ In the study by Taieb et al, only 5.2% of patients received L3, but unfortunately, there are no data concerning survival or the regimen used in L3.^13^ In the study by Sawada et al, 32.7% of patients received L3 after progression on GnP (L1) and mFfx (L2), 13.5% with gemcitabine and erlotinib, 12.5% with S-1 monotherapy and 6.7% with other regimens.^19^ Survival in patients on L3 was not reported.

In our study, patients had a longer median OS of 16.8 months compared to 11.1 months in the princeps L1 study, thereby showing the efficacy of the Ffx regimen.^5^ We believe that the difference is mainly explained by the selection of a subset of patients who were able to receive at least three lines of treatment, not only due to the efficacy of previous lines but also because improved supportive care over the past two decades maintains patients in better general health. In addition, the natural history of a slow-growing disease may have played a role in some patients.^5,21^ Patients with pancreatic cancer and lung-only metastasis are known to have a better prognosis. However, in our cohort, they represented less than 5.0%, which does not allow for accurate survival analysis.^32^

In a retrospective study, Bachet et al specifically analyzed L2 and L3 for aPDAC in 117 patients, of whom 21% had three chemotherapy lines or more.^21^ Survival data were available, but as this study was conducted between 1997 and 2006, before the development of Ffx or gemcitabine-nab-paclitaxel, there are no data for patients on these regimens. Median TTP was 2.5 months, and median OS from the beginning of L3 was 7.2 months longer than in our study (4.2 months). The retrospective single-center study conducted by Lu et al focused specifically on patients who had received at least three lines of treatment, with a total of only 72 patients enrolled between 2013 and 2023.^33^ For L3, median OS (6.9 months *vs* 4.2 months in our cohort) and PFS (4.4 months vs 2.2 months) were higher than in our study. This was probably related to the lower proportion of patients with liver metastases in their study (68.1% vs 78.3% in our cohort). More than one-third of the patients in our cohort received a fourth line of treatment, which is higher than the 7% reported by Abrams *et al.*^34^ This may result from differences in practices between centers and physicians in decisions regarding active treatment vs BSC alone.^34,35^ In the study by Palmieri et al, a high proportion of patients with gastrointestinal cancer were given chemotherapy in the month prior to death (26.3%), a percentage similar to that in our study (30.3%).^36^ The French National Cancer Institute set a target of less than 15% of cancer patients receiving chemotherapy in the last month of life as a quality and safety criterion of cancer care.

Approximately 20% of the patients in our cohort experienced grade 3 or 4 adverse events (no treatment-related deaths occurred), which is lower than the 47.2% rate reported in the study by Lu et al with chemotherapy.^33^ Given the limited efficacy of L3 chemotherapy in aPDAC and the significant risk associated with these adverse events, it is essential to discuss them with the patient before deciding to start L3 chemotherapy.

The criterion “no depletion of therapeutic resources,” defined as patients who had not used up all options for conventional drugs (5-fluorouracil, oxaliplatin, irinotecan, gemcitabine, and taxanes), emerged as a strong predictor of OS prior to starting L3. There are two treatment strategies, the first uses many chemotherapies (triplet) in L1 to improve efficacy, while the second uses fewer molecules (doublet) to keep options in reserve for subsequent treatment lines. However, the risk of a rapid deterioration in the general health of patients with an aggressive disease, such as aPDAC, may compromise the use of subsequent chemotherapy, and may thus justify the use of a more “aggressive” treatment in L1. We confirm these two treatment strategies in our study regarding the Sankey diagram since patients were evenly distributed between the chemotherapy triplet (Ffx) and a gemcitabine-based regimen (alone or in combination) in the first-line setting. Concerning L2 after a triplet regimen in L1, no study has demonstrated the superiority of a doublet over a single agent. In the GEMPAX study, PFS was significantly better in patients treated with a combination of chemotherapy agents (gemcitabine and paclitaxel) than in those treated with gemcitabine alone, at 3.1 vs 2.0 months, respectively (HR = 0.64 [0.47-0.89]) as in Zaibet et al, 3.5 vs 2.3 months (HR = 0.53 [0.43-0.65]).^20,37^ However, the combination therapy in the GEMPAX trial did not significantly improve median OS, at 6.4 months vs 5.9 months, respectively (HR = 0.87 [0.63-1.20]). This might suggest that a sequential strategy using gemcitabine monotherapy for L2 followed by paclitaxel for L3 could allow more treatment lines to be utilized effectively.^20^ This strategy was observed for a part of our cohort with the prescription of taxane mainly after gemcitabine alone and to a lesser extent after 5FU platinum or 5FU irinotecan.

In a multivariate analysis, Lee et al showed that after L1 with GNp, a high NLR (HR = 1.58 [1.05-2.39]) and a short L1 TTP (HR = 1.57 [1.05-2.36]) were independent predictors of poor L2 OS.^18^ In our study, L1 and L2 duration were also associated with OS and PFS in L3. In 2020, Sawada et al included 104 patients treated with modified Ffx in L2 after L1 GNp, and reported that a CEA level ≤ 10 ng/mL and Glasgow prognostic score (GPS) = 0 were independent prognostic factors for OS and PFS.^19^ We did not include NLR, CEA and GPS in our model because too many data were missing. Our study confirmed the prognostic value of common criteria previously described in L1 and L2, such as ECOG-PS, resection of the primary tumor, and the duration of the prior treatment lines.^3,5,6,18,19^ The latter probably reflects both the chemosensitivity of the disease and the weak aggressiveness of the tumor. To our knowledge, only one study has investigated predictors of PFS/OS in L2 after L1 with Ffx, and this study also reported ECOG-PS as a predictor of PFS in L2.^38^ Our study allowed us to assess survival factors in L3 regardless of the treatments received in L1 and L2 by identifying “universal” prognostic factors in L3. We then developed an online scoring tool based on simple and clinically relevant criteria that could be useful in clinical practice. However, to improve the prognosis of patients with aPDAC, it is essential to develop new effective drugs and therefore to enroll patients in clinical trials.

The main limitation of our study is the retrospective nature of the data and the large amount of missing information, particularly regarding biological parameters. A validation cohort would be necessary to confirm the robustness of our score in a prospective setting. Another significant limitation of this study is the absence of “control” patients who did not receive three lines of treatment. Such a control group would have enabled us to better assess the factors associated with the benefit of L3 compared with exclusive BSC. However, in the absence of randomization, this control group would probably skew the results as it would consist mainly of the most frail patients with the most aggressive tumors and thus ineligible for L3. Nevertheless, our score helps to determine expected survival with the L3 treatment and could help physicians in the decision-making process in routine practice. This cohort was exclusively French, which could limit the use of the online tool in other countries, since therapeutic strategies could differ from one country to another, as could patients’ characteristics. It would therefore be interesting to validate our tool in L3 aPDAC populations in other countries. The main strength of this study is the size of the population, and the wide range of centers included.

In conclusion, we created a tool based on robust predictors of outcomes in patients with aPDAC treated with L3. The tool could help physicians to determine the survival benefit of implementing L3. Another remaining challenge in aPDAC is to determine the optimal sequencing of chemotherapies in the second and third lines.

## Supplementary Material

oyaf125_suppl_Supplementary_Tables_1-2

oyaf125_suppl_Supplementary_Figures_1

oyaf125_suppl_Supplementary_Figures_2

oyaf125_suppl_Supplementary_Figures_3

oyaf125_suppl_Supplementary_Figures_5

oyaf125_suppl_Supplementary_Figures_4

## Data Availability

David Tougeron and Camille Evrard had full access to all the data in the study and took responsibility for the integrity of the data and the accuracy of the data analysis. Data are available upon reasonable request.
