# Supplementary material for: Predictive and prognostic factors of efficacy of third-line chemotherapy in patients with unresectable pancreatic cancer: a cohort-based study
Source: Oncologist. 2025 Jun 14;30(6):oyaf125. doi: 10.1093/oncolo/oyaf125 (PMC12166115; doi:10.1093/oncolo/oyaf125)
Supplement: oyaf125_suppl_Supplementary_Tables_1-2 [file oyaf125_suppl_supplementary_tables_1-2.docx]

**Supplementary Table 1: Patients’ and tumor characteristics at cancer diagnosis**

**(n=202).**

| **Clinical characteristics** | N = 202 |
| --- | --- |
| Male (n, %) | 103 (51.0%) |
| Age (years, median [Q1; Q3]) | 63.1 [55.8; 70.8] |
| BMI before disease (kg/m^2^, median [Q1; Q3], NA=33) | 26.2 kg/m^2^ [23.3; 29.4] |
| Loss of weight at diagnosis (%, median [Q1; Q3], NA=38) | 10.0 [5.0; 16.0] |
| ECOG PS (n, %)  0-1  ≥ 2 | 159 (90.9%)  16 (9.1%) |
| Diabetes mellitus (n, %) | 37 (18.3%) |
| **Biological characteristics** |  |
| NLR (median [Q1; Q3], NA=68) | 3.3 [2.0; 6.4] |
| AST (<IU/L, median [Q1; Q3], NA=63) | 32.0 [19.0; 67.0] |
| ALT (IU/L, median [Q1; Q3], NA=64) | 37.0 [24.0; 97.5] |
| ALP (IU/L, median [Q1; Q3], NA=64) | 126.5 [72.5; 270.0] |
| GGT (IU/L, median [Q1; Q3], NA=73) | 105.0 [40.0; 489.0] |
| Total bilirubin (umol/L, median [Q1; Q3], NA=47) | 10.0 [5.0; 36.5] |
| LDH (IU/L, median [Q1; Q3], NA=129) | 292.0 [254.0; 358.0] |
| Albumin (g/L, median [Q1; Q3], NA=70) | 40.0 [33.0; 43.0] |
| CEA (IU/L, median [Q1; Q3], NA=62) | 24.50 [4.0; 62.3] |
| CA 19-9 (IU/L, median [Q1; Q3], NA=50) | 562.0 [75.0; 3273.0] |
| **Tumor characteristics** |  |
| Tumor location (n, %, NA=3)  Head  Body  Tail  Multiple | 105 (52.8%)  48 (24.1%)  31 (15.6%)  15 (7.5%) |
| Resectable without metastasis (n, %)  Synchronous metastasis (n, %)  Locally advanced (n, %) | 34 (16.8%)  140 (69.3%)  28 (13.9%) |
| Number of metastatic sites* (NA=62, n, %)  1  >1 | 98 (70.0%)  42 (30.0%) |
| Location of metastasis* (NA=62, n, %)  Peritoneum  Liver  Lung | 36 (25.7%)  99 (70.7%)  21 (15.0%) |

NA: not attributed. SD: standard deviation. BMI: body mass index. ECOG PS: Eastern Cooperative Oncology Group Performance Status. NLR: neutrophil to lymphocyte ratio. ALT: alanine aminotransferase. AST: aspartate aminotransferase. ALP: alkaline phosphatase. GGT: gamma glutamyl transferase. LDH: lactate dehydrogenase. CEA: carcinoembryonic antigen. CA 19-9: carbohydrate antigen 19-9; umol/L: 10⁻⁶moles per liter; IU/L: international units per liter; [Q1; Q3]: first and third quartiles.

**Supplementary Table 2: Univariate analysis for predictive and prognostic factors progression-free survival and overall survival with L3.**

|  | **Progression-free survival**  **HR [95%CI]** | **Overall survival**  **HR [95%CI]** |
| --- | --- | --- |
| Age at start of L3 (Years) | 0.99 [0.98; 1.01] | 0.99 [0.98; 1.01] |
| Sex (Male versus Female) | 1.33 [1.01; 1.84] | 1.28 [0.96; 1.77] |
| Surgery of primary tumor (Yes versus No) | 0.56 [0.41; 0.78] | 0.54 [0.37; 0.76] |
| L1 chemotherapy (Folfirinox versus Other) | 1.22 [0.93; 1.65] | 1.06 [0.78; 1.47] |
| Depletion of therapeutic resources* (Yes versus No) | 1.37 [1.03; 1.92] | 1.36 [0.99; 1.96] |
| ECOG PS at start of L3 (0-1 versus 2-3) | 0.60 [0.45; 0.83] | 0.57 [0.42; 0.76] |
| ECOG PS at start of L3  0  1  2  3 | Ref.  0.92 [0.56 ; 1.52]  0.99 [0.35 ; 2.77]  2.34 [0.59 ; 9.22] | Ref.  1.74 [1.03; 2.95]  3.37 [1.13; 10.04]  16.04 [3.73; 69.03] |
| Lung metastasis at start of L3 (Yes versus No) | 0.94 [0.69; 1.25] | 1.17 [0.85; 1.57] |
| Peritoneum metastasis at start of L3 (Yes versus No) | 1.33 [0.94; 1.82] | 1.36 [0.97; 1.82] |
| Liver metastasis at start of L3 (reference: with other) |  |  |
| Isolated | 1.35 [0.97; 1.91] | 0.84 [0.59; 1.30] |
| No | 0.58 [0.43; 0.83] | 0.68 [0.49; 0.92] |
| Logarithm of L1 duration (Months) | 0.97 [0.80; 1.17] | 1.02 [0.84; 1.26] |
| Logarithm of L2 duration (Months) | 0.63 [0.51; 0.80] | 0.57 [0.44; 0.71] |

HR: hazard ratio; 95%CI: 95% confidence interval; ECOG PS: Eastern Cooperative Oncology Group Performance Status; L1: first-line treatment; L3: third-line treatment; Ref: reference

*defined as a patient who had already received 5-fluoruracil, oxaliplatin, irinotecan, gemcitabine and taxane at the beginning of L3.
