# Supplementary material for: Predictive and prognostic factors of efficacy of third-line chemotherapy in patients with unresectable pancreatic cancer: a cohort-based study
Source: Oncologist. 2025 Jun 14;30(6):oyaf125. doi: 10.1093/oncolo/oyaf125 (PMC12166115; doi:10.1093/oncolo/oyaf125)
Supplement: oyaf125_suppl_Supplementary_Figures_1 [file oyaf125_suppl_supplementary_figures_1.docx]

**Supplementary Figure 1: Flow-chart of population according to chemotherapy setting in L1, L2, and L3**

**1A: Patients with 5FU-based chemotherapy in L1. 1B: Patients with Gem-based chemotherapy in L1.**

1A :


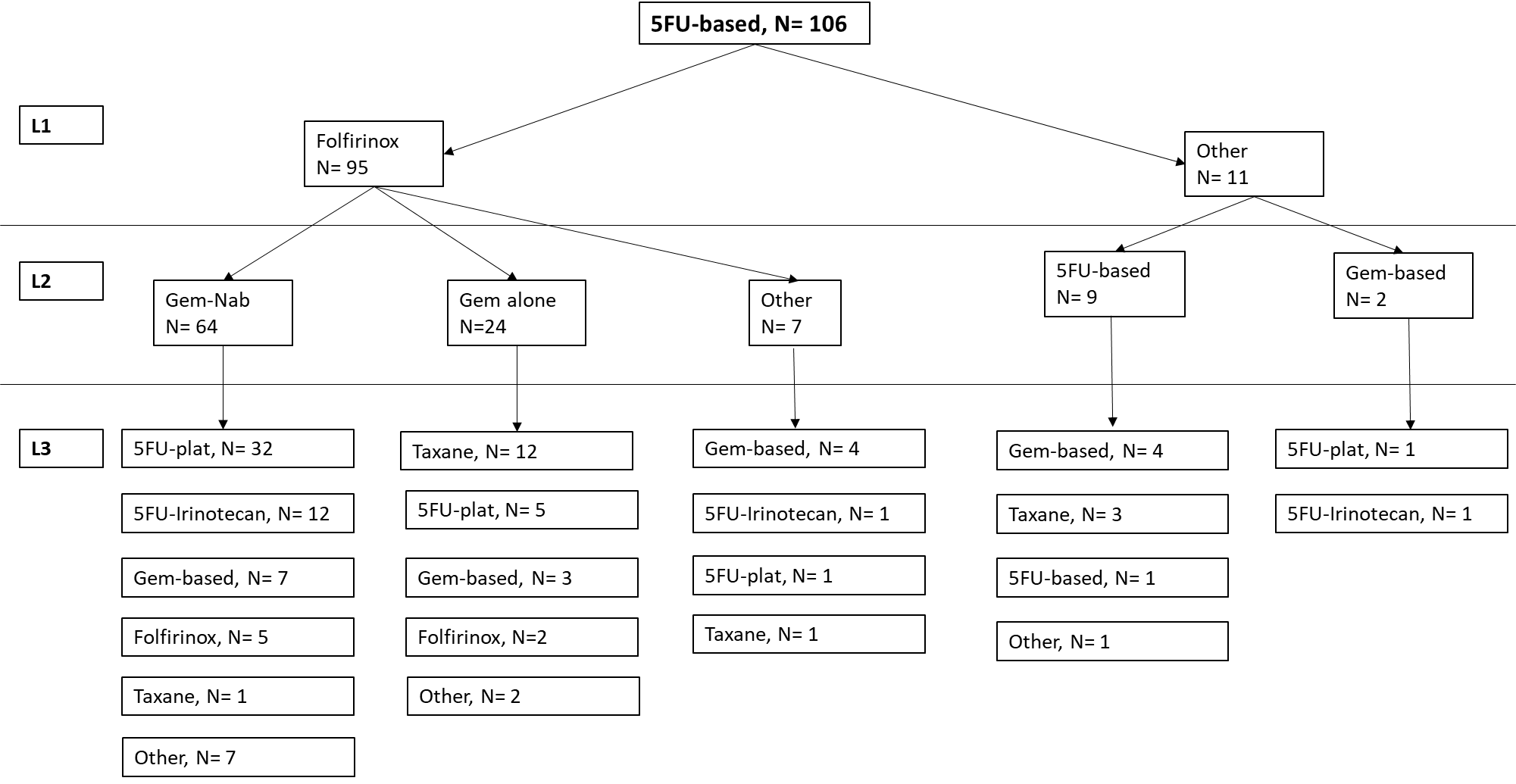


Legend: L1: first line; L2: second line; L3: third line; 5 FU: 5Fluorouracil; Gem-Nab: gemcitabine and nab-paclitaxel; 5FU-Iri: 5Fluorouracil and irinotecan or 5Fluorouracil and naliri or capecitabine and irinotecan; 5FU-plat: 5Fluorouracil and platinum salt; Gem-plat: gemcitabine and platinum salt; Ffx: folfirinox; Gmz: gemcitabine.


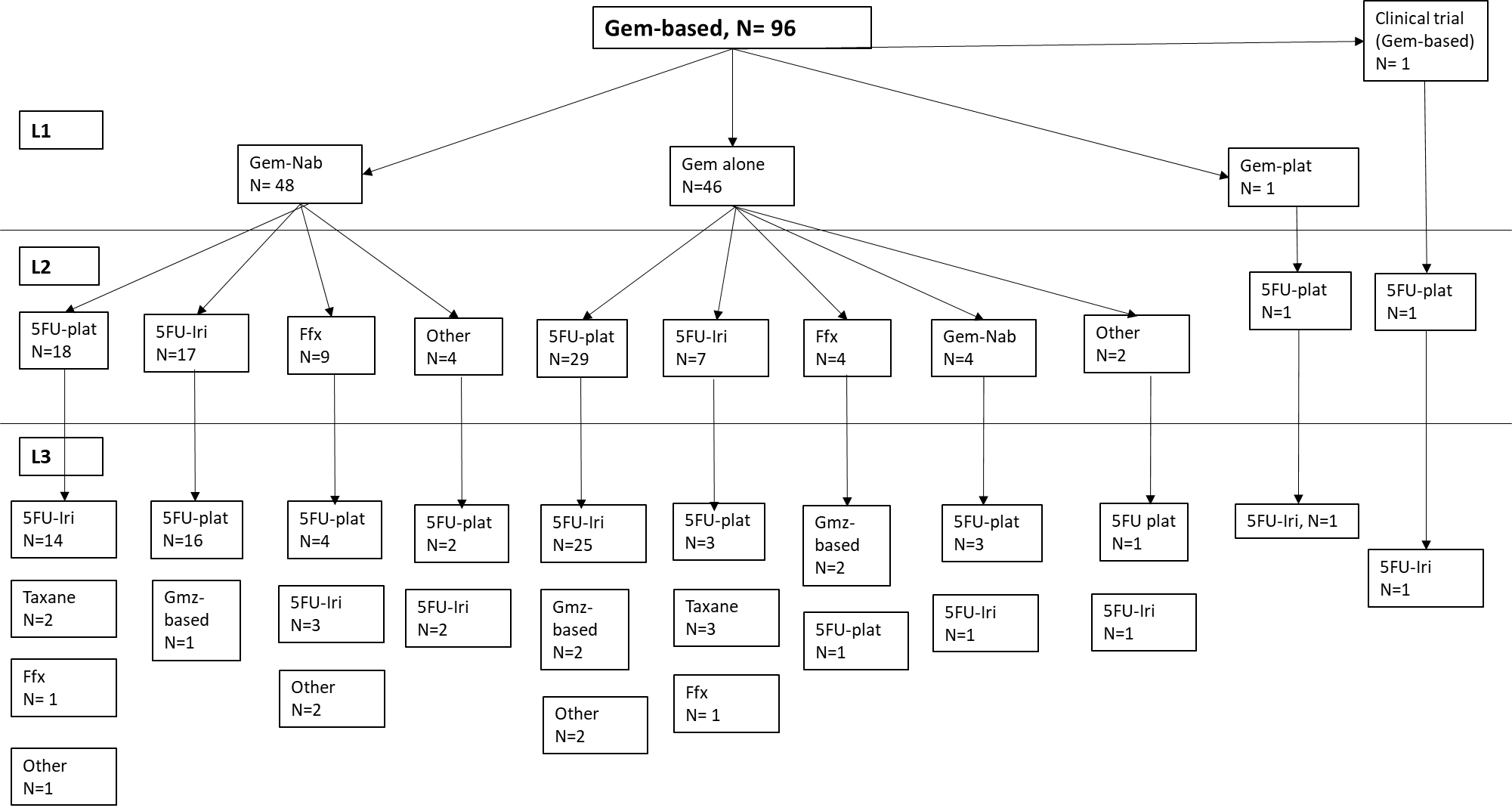
 Legend: L1: first line; L2: second line; L3: third line; 5 FU: 5Fluorouracil; Gem-Nab: gemcitabine and nab-paclitaxel; 5FU-Iri: 5Fluorouracil and irinotecan or 5Fluorouracil and naliri or capecitabine and irinotecan; 5FU-plat: 5Fluorouracil and platinum salt; Gem-plat: gemcitabine and platinum salt; Ffx: folfirinox; Gmz: gemcitabin

1B :
