# Supplementary material for: Predictive and prognostic factors of efficacy of third-line chemotherapy in patients with unresectable pancreatic cancer: a cohort-based study
Source: Oncologist. 2025 Jun 14;30(6):oyaf125. doi: 10.1093/oncolo/oyaf125 (PMC12166115; doi:10.1093/oncolo/oyaf125)
Supplement: oyaf125_suppl_Supplementary_Figures_2 [file oyaf125_suppl_supplementary_figures_2.docx]

**Supplementary Figure 2: Survival in first-line therapy**

2A: Progression-free survival in first-line therapy; 2B: Overall survival in first-line therapy

2A :

Median PFS = 6.1 months months

No. At Risk :

202 136 97 52 32 16 10 7 6

Progression-free survival

Months

2B :

Overall survival

Median OS = 16.8 months months

No. At Risk :

202 202 198 182 152 121 85 60 43

Months
