# Supplementary material for: Predictive and prognostic factors of efficacy of third-line chemotherapy in patients with unresectable pancreatic cancer: a cohort-based study
Source: Oncologist. 2025 Jun 14;30(6):oyaf125. doi: 10.1093/oncolo/oyaf125 (PMC12166115; doi:10.1093/oncolo/oyaf125)
Supplement: oyaf125_suppl_Supplementary_Figures_3 [file oyaf125_suppl_supplementary_figures_3.docx]

**Supplementary Figure 3: Survival in second-line therapy**

3A: Progression-free survival in second-line therapy; 3B: Overall survival in second-line therapy

3A:

Median PFS = 3.5 months

Progression-free survival

Months

No. At Risk :

202 114 54 27 13 7 4 3 3

3B:

Months

Median OS = 9.1 months months

Overall survival

No. At Risk :

202 193 153 100 67 43 31 21 16
