# Supplementary material for: Predictive and prognostic factors of efficacy of third-line chemotherapy in patients with unresectable pancreatic cancer: a cohort-based study
Source: Oncologist. 2025 Jun 14;30(6):oyaf125. doi: 10.1093/oncolo/oyaf125 (PMC12166115; doi:10.1093/oncolo/oyaf125)
Supplement: oyaf125_suppl_Supplementary_Figures_5 [file oyaf125_suppl_supplementary_figures_5.docx]

**Supplementary Figure 5: Example of estimated survival at L3 with our online calculator**

**Supplementary Figure 5A: case 1**

A 77-year-old man with synchronous metastatic pancreatic cancer without primary tumor resection, who received first-line gemcitabine-nab-paclitaxel (5.1 months of stable disease) and then second-line FOLFOX (2.2 months of stable disease). At L3, he had lung and liver metastases, and peritoneal carcinomatosis. Using our online calculator, his predicted PFS and OS with L3 would be 3.0 months and 4.5 months, respectively, suggesting that BSC may be the best strategy for this patient.

**Supplementary Figure 5B: case 2**

A 59-year-old woman with metachronous metastatic pancreatic cancer and primary tumor resection, treated with first-line FOLFIRINOX (partial response for 13.0 months) and second-line Gemcitabine-Nab-Paclitaxel (stable disease for 6.4 months), with the disease remaining limited to lung metastases. Her predicted PFS and OS with L3 were 8.9 months and 18.4 months, respectively, suggesting that L3 would be beneficial.

5A :

**
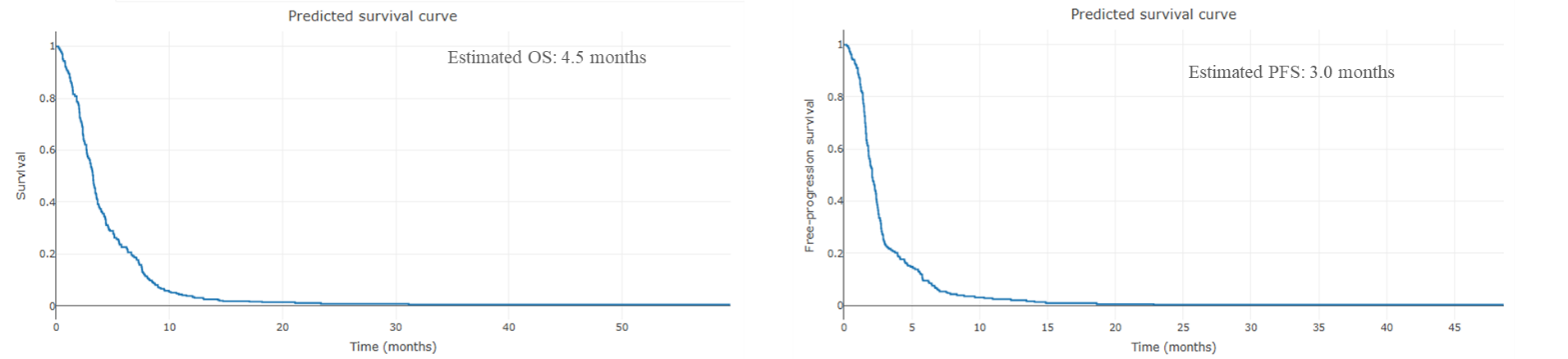
**

5B:

**
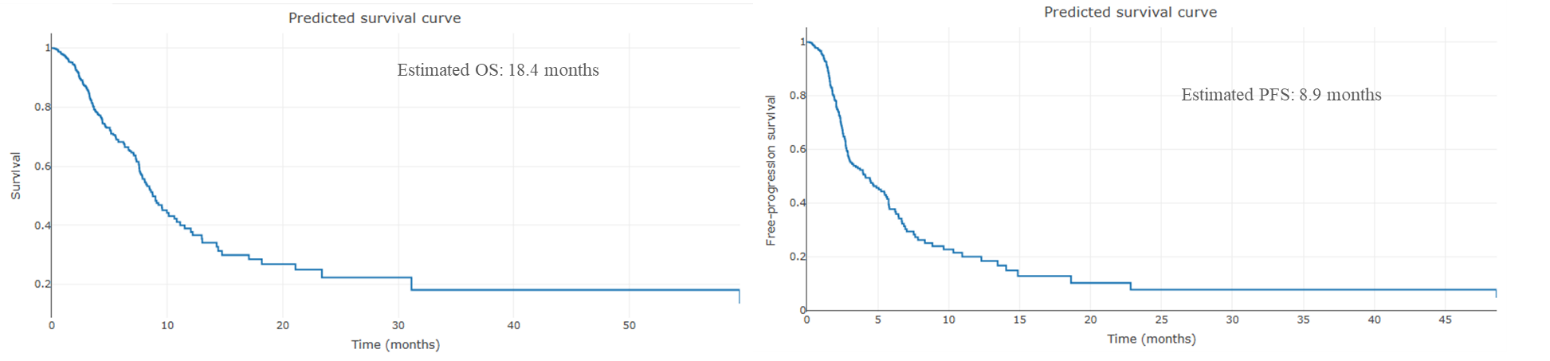
**

Legend: OS: overall survival; PFS: progression-free survival
