# Supplementary material for: Predictive and prognostic factors of efficacy of third-line chemotherapy in patients with unresectable pancreatic cancer: a cohort-based study
Source: Oncologist. 2025 Jun 14;30(6):oyaf125. doi: 10.1093/oncolo/oyaf125 (PMC12166115; doi:10.1093/oncolo/oyaf125)
Supplement: oyaf125_suppl_Supplementary_Figures_4 [file oyaf125_suppl_supplementary_figures_4.docx]

**Supplementary Figure 4 A, B, C and D: Calibration plots between observed and predicted survival with the 95% CI of the observed values**

Visual to assess the calibration, i.e. the agreement between predictions and observations.


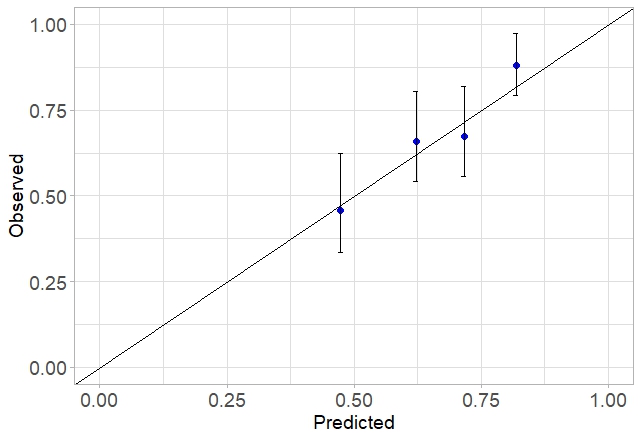

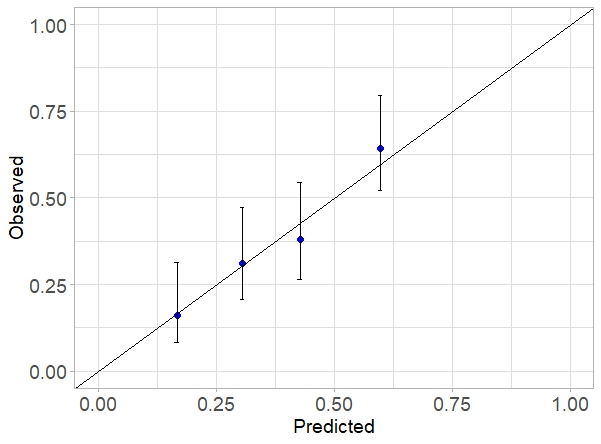


**A: Prediction of OS at 3 months**

**B: Prediction of OS at 6 months**


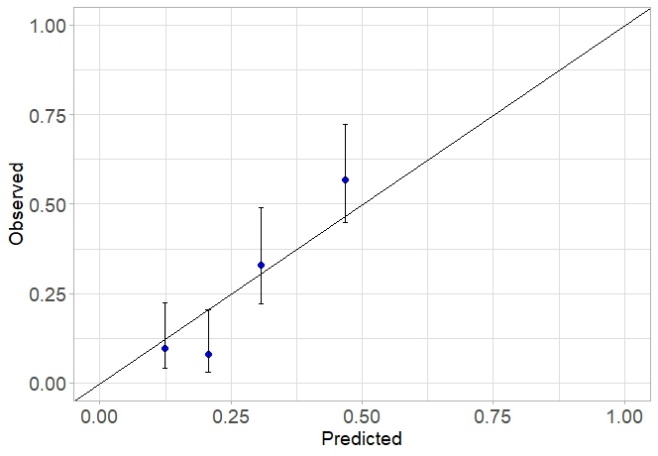

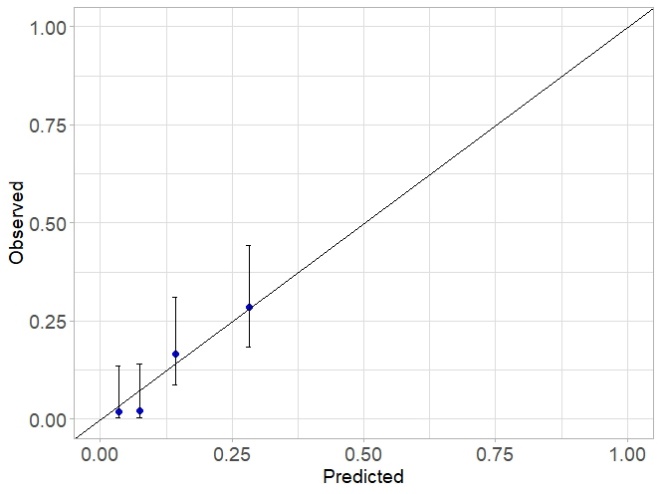


**C: Prediction of PFS at 3 months**

**D: Prediction of PFS at 6 months**

OS: overall survival; PFS: progression-free survival
